# Disparate biomechanical properties of the aorta in non‐aneurysmal and aneurysmal mice treated with angiotensin II

**DOI:** 10.14814/phy2.15410

**Published:** 2022-09-18

**Authors:** Sofie De Moudt, Jhana O. Hendrickx, Guido R. Y. De Meyer, Wim Martinet, Paul Fransen

**Affiliations:** ^1^ Laboratory of Physiopharmacology University of Antwerp Antwerp Belgium

**Keywords:** abdominal aortic aneurysm, aortic stiffness, contraction, vascular smooth muscle cells

## Abstract

In vivo angiotensin II (AngII)‐treatment is a widely used experimental model to induce cardiovascular disease and results in a high likelihood of abdominal aorta aneurysm (AAA) formation. This involves progressive and irreversible focal dilation of the abdominal aorta and induces adverse aortic connective tissue remodeling contributing to aortic wall stiffening through inflammation, elastin degradation, and collagen restructuring. Hence, the present study aimed to investigate how AAA formation in AngII‐treated mice affects aortic function and biomechanics. To this end, C57Bl/6J mice were treated with AngII (1000 ng/[kg.min]) or PBS infusion for 28 days. Peripheral blood pressure, echocardiography, and aortic pulse wave velocity were measured in vivo. Thoracic aorta rings were studied ex vivo in organ chambers, while aortic vascular smooth muscle cell (VSMC) phenotype was investigated histologically. We confirmed peripheral hypertension, cardiac hypertrophy, aortic stiffening, and increased VSMC proliferation and migration after AngII‐treatment. Abdominal aorta aneurysm formation was observed in 8/13 AngII‐treated mice. Ex vivo thoracic aortic rings of both aneurysmal and non‐aneurysmal AngII‐treated mice showed high isobaric aortic stiffness, endothelial dysfunction, heightened α_1_‐adrenergic contractility, and altered VSMC contractile calcium signaling. However, aortic biomechanics were differently affected, with heightened α_1_‐adrenoreceptor mediated aortic stiffening in non‐aneurysmal mice, whereas contraction‐dependent stiffening was impaired in aneurysmal mice. In conclusion, although aneurysmal and non‐aneurysmal 4‐week AngII‐treated mice displayed similar changes in aortic physiology, aortic biomechanics were dissimilarly affected.

## Graphical abstract

4‐Week angiotensin‐II (AngII)‐treated C57Bl/6J mice displayed similar in vivo cardiovascular disease characteristics and isometric reactivity changes of the ex vivo thoracic aorta, independent of the development of abdominal aortic aneurysms. However, biomechanical properties of the aorta were dissimilarly affected between aneurysmal and non‐aneurysmal AngII‐treated mice, highlighting the importance of investigating vasoactive signaling pathways in the context of active modulation of aortic biomechanical behavior.

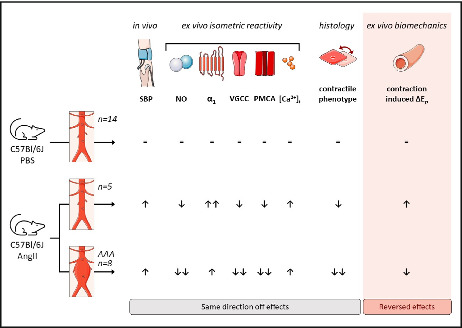

## 1 INTRODUCTION

As human life expectancy continues to grow, the incidence of age‐related cardiovascular diseases is rising. According to the World Health Organization, cardiovascular disease has long since become the leading cause of death, resulting in an estimated 17.9 million deaths each year, which represents over 30% of global death (World‐Health‐Organization, 2021). Arterial stiffening – defined as the impaired capacity of the large elastic arteries to smoothen pulsatile blood flow (Safar et al., 2014) – results in increased cardiac afterload, reduced coronary perfusion pressure and pulsatile strain on the microcirculation. As such, arterial stiffness has gained much recognition as a hallmark and independent predictor of cardiovascular disease (Laurent et al., 2003; Mattace‐Raso et al., 2006; Willum‐Hansen et al., 2006).

Angiotensin II (AngII) is the active peptide hormone of the renin angiotensin system (Wong, 2016). It was discovered in 1940 as a potent vasoconstrictor (Page & Helmer, 1940), although pleiotropic effects on the vascular system have since been described. High circulating levels of AngII are related to hypertension, renal insufficiency, and cardiac fibrosis (Wong, 2016). Therefore, in vivo AngII‐treatment is a commonly used experimental model to induce cardiovascular disease in small animal models (Murali Krishna et al., 2020). It is considered a strong stimulus, which affects the structural, molecular, functional, and biomechanical properties of the arterial wall (Eberson et al., 2015; Fransen et al., 2016; Leloup et al., 2018; Majeed et al., 2014). Most of the classical AngII symptoms (i.e., hypertension, thirst) are mediated by calcium/IP_3_ signaling after AngII receptor 1 (AT1) binding on vascular smooth muscle cells (VSMC) (Wong, 2016). This receptor is predominantly expressed on resistance arteries, whereas expression in the mouse thoracic aorta is low and no direct aortic contraction upon AngII stimulation is elicited (Fransen et al., 2016; Russell & Watts, 2000; Zhou et al., 2003). Of note, slight phasic contractions were reported in mouse aortic tissue upon high‐dose AngII stimulation, which were increased upon chronic AngII infusion (Spronck et al., 2021). These contractions were not of a magnitude expected to affect the contractile tone of the in vivo aorta, however. Therefore, the hypertensive response to circulating AngII is – among other mechanisms (Owens 3rd et al., 2010) – the result of increased peripheral resistance (Fransen et al., 2016), and its role in blood pressure control is underlined by the severe hypotensive phenotype of AT1a knockout mice (Chen et al., 2009).

Despite the absence of a direct AngII‐induced aortic contractile response, our research group previously showed that 4‐week AngII‐treatment affects active arterial reactivity of both aorta and femoral artery in a vascular bed‐specific manner (Fransen et al., 2016). The 4 week AngII‐treated mouse aorta was sensitized to depolarization‐induced contraction and to subsequent blockade of voltage‐gated calcium channels. In the femoral artery however, a desensitization to depolarization‐induced contractions was observed. We further performed ex vivo biomechanical studies of the aorta of 1 week AngII‐treated mice, and could demonstrate elevated aortic stiffness through active mechanisms involving impaired basal nitric oxide (NO) production leading to VSMC depolarization, even though no increase in blood pressure was observed after 1 week treatment (Leloup et al., 2018).

We previously reported that aortic VSMC contraction significantly alters the biomechanical properties of mouse aortic tissue (Leloup et al., 2019), which was recently confirmed for tissue specimens from the human descending thoracic aorta (Franchini et al., 2022). Aortic biomechanical properties and the role of VSMC contraction in aortic stiffness regulation were also shown to be altered during aging (Amabili et al., 2020, 2021; De Moudt et al., 2022b). Following these mechanical characterizations of healthy aortic tissue upon VSMC activation, we therefore now aim to provide improved insight in the biomechanical design of aortic tissue during cardiovascular disease development. The present study offers a biomechanical investigation of 4‐week AngII‐treated mice, at which time a distinct hypertensive phenotype is present. We hypothesize that chronic exposure to this increased blood pressure will induce pronounced changes on the VSMC level, beyond the effects of reduced NO bioavailability.

## 2 MATERIAL AND METHODS

The data that support the findings of this study are available from the corresponding author upon reasonable request.

### 2.1 Laboratory animals and tissue collection

All animal experiments were approved by the Ethical Committee of the University of Antwerp and conducted in accordance to the Guide for the Care and Use of Laboratory Animals, published by the National Institutes of Health (NIH Publication No. 85–23; Revised, 1996). Male C57Bl6/J mice (000664, The Jackson Laboratory) were purchased at the age of 8 weeks, and were housed in the animal facility of the University of Antwerp, with a 12 h/12 h light–dark cycle and free access to water and standard chow. At the age of 22 weeks, C57Bl6/J mice (*n* = 16) were placed under general anesthesia (2% isoflurane) and osmotic minipumps (ALZET, model 2004) were implanted subcutaneously for 28‐day continuous AngII (1000 ng/[kg.min], Sigma Aldrich) infusion. Control mice (*n* = 14) were similarly implanted with osmotic minipumps containing sterile phosphate buffered saline (PBS). After 24–28 day treatment, mice were placed under deep anesthesia (pentobarbital sodium, 75 mg/kg ip; Sanofi, Belgium), and mice were euthanized by perforation of the diaphragm. The thoracic aorta and heart were carefully removed and the aorta was stripped of adherent tissue. Next, aortic rings of 2 mm width were cut starting at the diaphragm, in the distal to proximal direction. Aortic rings were numbered TA0 to TA5 (proximal to distal thoracic aorta) as illustrated in Figure S1. Of these, two segments were used for ex vivo isometric reactivity studies (TA3 and TA4), two segments for ex vivo assessment of biomechanical aortic properties (TA1 and TA2), and one segments was fixed in 4% formaldehyde for histological staining (TA0). Heart weight was recorded and the heart was longitudinally cut in half for formaldehyde fixation.

### 2.2 In vivo cardiovascular characterization

At day 23 of AngII‐treatment, in vivo cardiovascular parameters were assessed. Peripheral blood pressure was measured with the CODA tail‐cuff method as previously described (Fransen et al., 2016). In brief, a pressure‐volume sensor was attached to the tail of conscious restrained mice distally to an occluding cuff. Systolic and diastolic blood pressure were measured three times over a period of 1.5 days, of which the final measurement was used. Next, transthoracic echocardiograms were acquired in anesthetized mice (1.5–2.5% isoflurane v/v [Forene, Abbvie]) using high frequency ultrasound (Vevo2100, Visualsonics). Heart rate (from the Vevo2100 ECG reading) was maintained at 500 ± 50 beats/min (by isoflurane titration) and body temperature between 36–38°C. M‐mode images were obtained for left ventricular (LV) function evaluation on short axis view, including measurement of interventricular septum (IVS) thickness, LV posterior wall thickness and LV lumen diameter on three consecutive respiratory cycles. Fractional shortening, ejection fraction, LV mass, and stroke volume were calculated. On a four‐chamber view, diastolic heart function parameters were assessed using pulsed wave Doppler analysis of blood flow through the mitral valve, which allows for measurement of the E wave, A wave, isovolumic relaxation time and deceleration time, and calculation of the E/A ratio. Finally, abdominal aorta pulse wave velocity (aPWV) was measured as described by Di Lascio et al. (2014). In short, B‐mode images of aortic diameter and pulsed wave Doppler analysis of velocity were acquired and averaged over several cardiac cycles. aPWV was calculated as dV/2dln(D) (with dV, velocity change; and dln(D), the variation of the natural logarithm of diameter).

### 2.3 Isometric reactivity studies

Two 2‐mm aortic rings of two mice each (one PBS‐ and one AngII‐infused, chosen at random) were mounted between two parallel wire hooks in four 10‐ml organ bath containing Krebs‐Ringer solution (composition [mM]: NaCl 118; KCl 4.7; CaCl_2_ 2.5; KH_2_PO_4_ 1.2; MgSO_4_ 1.2; NaHCO_3_ 25; CaEDTA 0.025; glucose 11.1) and were studied in parallel. The solution was continuously heated to 37°C and aerated with a 95% O_2_/5% CO_2_ gas mixture to maintain a pH of 7.4. A preload of 20 mN was applied to approximate normal physiological stretch at a mean calculated blood pressure of 100 mmHg (De Moudt et al., 2017), and isometric contractions and relaxations were measured by means of a Statham UC2 force transducer (Gould, USA). In the first aortic ring, contractions were induced by concentration‐response stimulation with α_1_‐adrenergic agonist phenylephrine (PE, 3 nM to 10 μM), followed by endothelium‐dependent relaxations by concentration‐response stimulation with acetylcholine (ACh, 3 nM to 1 μM). Next, 300 μM N‐Ω‐Nitro‐L‐arginine methyl ester hydrochloride (L‐NAME, NOS blocker) was added to the organ bath followed by endothelium‐independent relaxations using concentration‐response stimulation with diethylamine NONOate (DEANO, 0.3 nM–10 μM). Basal NO levels were quantified as the relative difference in PE‐induced contractile force in the absence and presence of L‐NAME. In the second aortic ring, Krebs‐Ringer solution was replaced with a solution lacking calcium (0Ca Krebs) to avoid extracellular calcium influx, and a transient sarcoplasmic (SR)‐mediated contraction was induced by 2 μM PE as previously described (Fransen et al., 2015). Thereafter, extracellular calcium is re‐introduced to evoke a tonic contraction, followed by addition of 35 μM diltiazem, to completely block calcium entry through voltage‐gated calcium channels (VGCC). All concentration‐response curves were fitted with a non‐linear 4‐parameter equation, to obtain values for maximal effect and half‐maximal effective or inhibitory concentration (EC_50_ or IC_50_). Aortic rings were rotated between organ baths to exclude possible setup effects, and alternately assigned to the first or second protocol.

### 2.4 Isobaric measurement of aortic stiffness

Two 2‐mm aortic rings of two mice each (one PBS‐ and one AngII‐infused, chosen at random) were mounted in a Rodent Oscillatory Tension Set‐up for Arterial Compliance (ROTSAC) between two parallel wire hooks in four 10‐ml organ chambers containing Krebs‐Ringer solution and were studied in parallel. The upper wire hook was connected to a force‐length transducer, and segments were continuously stretched between alternating preloads corresponding to a calculated “systolic” and “diastolic” transmural pressure (Laplace law) at a physiological frequency of 10 Hz to mimic the physiological heart rate in mice (600 bpm) (Leloup et al., 2016). At any given pressure, calibration of the upper hook allowed for the calculation of the diastolic and systolic vessel diameter (mm) and Peterson modulus (E_p_). E_p_ was defined as the pulse pressure divided by the relative diameter change (E_p_ = D_0_ × ΔP/ΔD). Aortic stiffness was always assessed in isobaric conditions, and measured at calculated oscillating pressures of 60–100, 80–120, 100–140 and 120–160 mmHg. Isobaric aortic stiffness was assessed in non‐contracted (Krebs‐Ringer) and contracted (2 μM PE with/without 300 μM L‐NAME) conditions. Furthermore, VGCC were blocked in PE‐preconstricted aortic rings using 35 μM diltiazem, and contractile tone was negated using a 0 Ca Krebs environment.

### 2.5 Histology

Aortic and cardiac tissues were fixed for 24 h in 4% formalin solution (BDH Prolabo), and subsequently dehydrated in 60% isopropanol (BDH Prolabo), followed by paraffin‐embedding. Aortic media thickness was measured on orcein‐stained sections of the aorta, which allows for accurate assessment of the inner and outer border of the media layer. Orcein staining was further used to assess elastin content and for counting elastin breaks and elastic laminae. The latter is assessed as the average of 8 measurements across the aortic wall. VSMC phenotype was assessed using immunofluorescent myocardin staining (Sigma, sab4200539), and immunohistochemical staining for α‐smooth muscle actin (α‐SMA, Sigma, F‐3777) and proliferating cell nuclear antigen (PCNA, Biorad, MCA1558F). Cardiac fibrosis was assessed using trichrome Masson staining and cardiac hypertrophy was quantified on the cellular level using an anti‐laminin (Novus Biologicals, nb300‐144) staining. Five images off each mouse were recorded for this measurement in different cross‐sectional regions of the heart, and cross‐sectional area of 20 cardiomyocytes was measured per image (final average of 100 measurements). Microscopic images were acquired with Universal Grap 6.1 software using an Olympus BX4 microscope or Celena S fluorescence microscope and quantified using ImageJ software.

### 2.6 Statistical analysis

Data is expressed as mean ± SEM, with n representing the number of biological replicates. All analyses were performed using GraphPad Prism (version 8, GraphPad Software) and a significance level of 5% was set to identify statistically significant changes. Normality of data was verified using the Kolmogorov–Smirnov test, and parametric testing was used when indicated.

**FIGURE 1:**
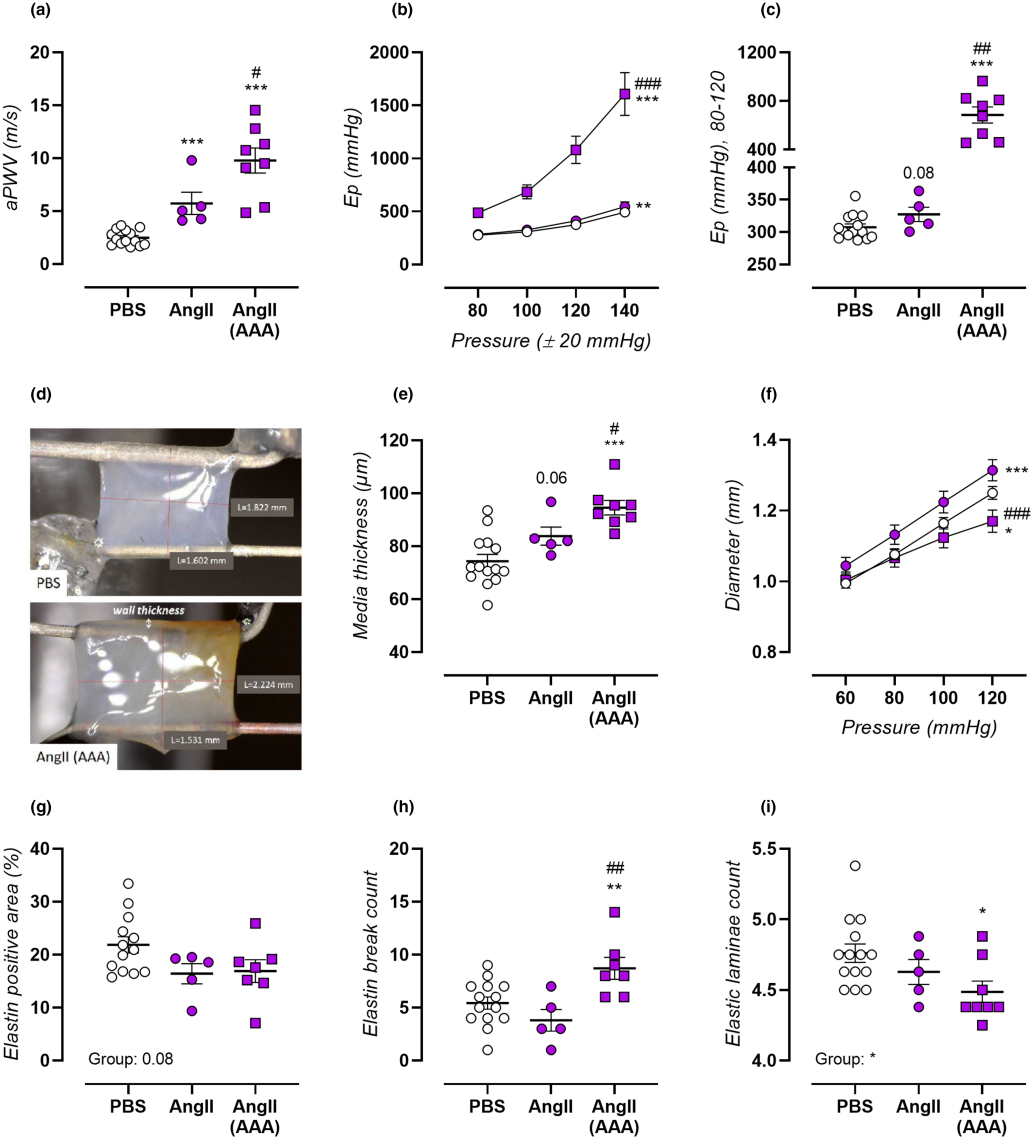
Increased aortic stiffness in AngII‐treated mice. Aortic stiffness was assessed in PBS‐treated control mice (*n* = 14, open circles), non‐aneurysmal 4‐week AngII‐treated mice (*n* = 5, filled circles), and aneurysmal 4‐week AngII‐treated mice (*n* = 8, filled squares). In vivo measurement of aortic pulse wave velocity (aPWV, a), was combined with ex vivo measurement of thoracic aorta Peterson modulus (E_p_). The latter was studied over a pressure range of 60–100 mmHg to 120–160 mmHg (b) and specifically shown at physiological 80–120 mmHg pressure (c). Representative pictures of mounted aortic segments of the PBS and AngII (AAA) groups are shown, indicating increased aortic wall thickness (arrow) in aneurysmal AngII‐treated mice (d). Medial wall thickness was measured on histological aortic sections (e) and isobaric aortic diameter was assessed in the ROTSAC set‐up. Histological orcein staining of the thoracic aorta was used to assess media elastin positive area (g), elastin break count (h) and elastic laminae count (i). Data are listed as mean ± SEM (b,f; *n* = 14, 5, 8) or each symbol represents a biological repeat (a,c,e, g–i). Statistical analysis using one‐way ANOVA (g,i) multiple *t*‐testing (a,c,e,h) or multiple two‐way ANOVA (b,f). Significance level compared to the PBS group is listed using *, and between AngII and AngII AAA groups using #. Overall one‐way ANOVA significance (bottom) or post‐hoc/*t*‐test/overall two‐way ANOVA significance (in graph) are listed. */#*p* < 0.05, **/##*p* < 0.01, ***/###*p* < 0.001.

Data was stratified according to the presence of an aneurysm. Due to high magnitude changes in aneurysmal AngII‐treated mice, the following statistical analysis approach was used:

- **Step 1:** Statistical analysis was performed using one‐way, two‐way, or three‐way ANOVA testing (depending on the data format), with post‐hoc testing using a Tuckey multiple testing correction.
- **Step 2:** The post‐hoc significance between the aneurysmal and non‐aneurysmal AngII‐treated groups was consulted.
- If no significant difference was observed (e.g., Figure 1g), the one‐way/two‐way/three‐way ANOVA testing was kept.
- If a significant difference was observed (e.g. Figure 1c), statistical analysis was continued with Step 3.
- **Step 3:** Statistical analysis was performed using multiple t‐testing, multiple one‐way, or multiple two‐way ANOVA testing (depending on the data format), which replaced the analysis from Step 1. This, to allow for an accurate assessment of the statistical significance in the non‐aneurysmal AngII‐treated group versus control.

## 3 RESULTS

### 3.1 AngII‐treatment results in prevalent aneurysm formation and peripheral hypertension

C57Bl6/J mice were treated with AngII or PBS infusion over a period of 28 days. During this period, 20% mortality was observed in AngII‐treated mice, whereas no mortality occurred in control animals (Figure S2a). AngII treatment also resulted in the development of an aneurysm in 8/13 mice which survived the treatment as observed during dissection (Figure S2b). Abdominal aortic aneurysms (AAA) were most frequently encountered, although in severe cases the aortic arch and/or thoracic aorta were sometimes affected as well. Due to high variability between aneurysmal and non‐aneurysmal AngII‐treated mice, all further data in this paper was stratified depending on the discovery of an AAA during dissection. AngII‐treatment further resulted in a significant decline in body weight in both non‐aneurysmal and aneurysmal AngII‐treated mice (Figure S2c), and induction of a clear hypertensive phenotype with elevated peripheral systolic, diastolic, and pulse pressure (Figure S2d–f) independent of the presence of an AAA.

### 3.2 AngII‐treatment induces cardiac hypertrophy

AngII‐treated mice displayed an increased heart/body weight ratio, with a 17% increase in non‐aneurysmal and a 36% increase in aneurysmal AngII‐treated mice compared to PBS‐treated control animals (Table S1), demonstrating more pronounced cardiac hypertrophy in aneurysmal AngII‐treated mice. Echocardiograms were used for further assessment of cardiac function (Table S1). This revealed clear LV hypertrophy (elevated IVS thickness, LV posterior wall thickness, LV mass/body weight ratio) with preserved stroke volume and ejection fraction. The hypertrophic response coincided with a non‐significant trend towards increased LV internal diameter (AngII, 10% dilation *p* = 0.07; AngII (AAA), 8% dilation *p* = 0.17), indicating eccentric cardiac hypertrophy. Diastolic function of AngII‐treated mice was also assessed, but no significant changes were detected. Histological analysis confirmed AngII‐induced cardiac hypertrophy on the cellular level, showing a significantly increased cardiomyocyte cross‐sectional area, and further revealed significant cardiac fibrosis due to AngII treatment. Both parameters were increased independent of AAA formation (Table S1) (representative images, Figure S3).

### 3.3 AngII‐treatment results in increased aortic stiffness, which was most pronounced in aneurysmal AngII‐treated mice

In vivo measurement of aortic stiffness revealed an significantly elevated aPWV of 5.7 ± 1.0 m/s (*p* = 1.70E‐04) in non‐aneurysmal, and severely elevated aPWV of 9.8 ± 1.2 m/s (*p* = 1.35E‐07) in aneurysmal AngII‐treated mice (Figure 1a). Ex vivo thoracic aorta stiffness was further assessed in isobaric conditions as E_p_ over a broad pressure range, with a significant increase in both aneurysmal and non‐aneurysmal AngII‐treated mice which is most pronounced at high distending pressure (Figure 1b). This confirms that in vivo elevated aPWV values represent vessel wall specific changes. At 80–120 mmHg, an increased E_p_ of 327 ± 11 mmHg (*p* = 0.08) was observed in non‐aneurysmal and 685 ± 66 mmHg (*p* = 2.42E‐07) in aneurysmal AngII‐treated mice compared to mice treated with PBS (307 ± 5 mmHg) (Figure 1c). The presence of an AAA therefore greatly increases AngII‐induced effects on thoracic aortic stiffness. This increased stiffness could for a large part be contributed to pronounced aortic wall hypertrophy, which was already visible macroscopically for aneurysmal AngII‐treated mice as an increased wall thickness (Figure 1d), and was further confirmed using histological measurement of aortic media thickness (Figure 1e). Isobaric baseline diameter was assessed ex vivo in a ROTSAC set‐up, and showed an increase in non‐aneurysmal AngII‐treated mice, indicating eccentric hypertrophy, whereas aneurysmal AngII‐treated mice had unaltered diameters at physiological pressure but displayed an impaired pressure‐dependent diameter dilation (Figure 1f). Orcein staining of thoracic aortic sections revealed a non‐significant trend to reduced percentage media elastin positive area in both AngII‐treated groups (*p* = 0.08) (Figure 1g). Furthermore, aneurysmal mice displayed a pronounced accumulation of elastin breaks (Figure 1h) and even a significantly reduced number of elastic laminae (Figure 1i), indicating marked restructuring of the aortic media extracellular matrix (representative image, Figure S4). Of note, no significant differences in absolute elastin area were observed between groups (PBS, 0.039 ± 0.003 mm^2^; AngII, 0.033 ± 0.005 mm^2^; AngII (AAA), 0.040 ± 0.005 mm^2^; *p* = 0.47).

### 3.4 At calculated 80–120 mmHg pressure, contraction‐dependent stiffening is elevated in non‐aneurysmal and impaired in aneurysmal AngII‐treated mice

**FIGURE 2:**
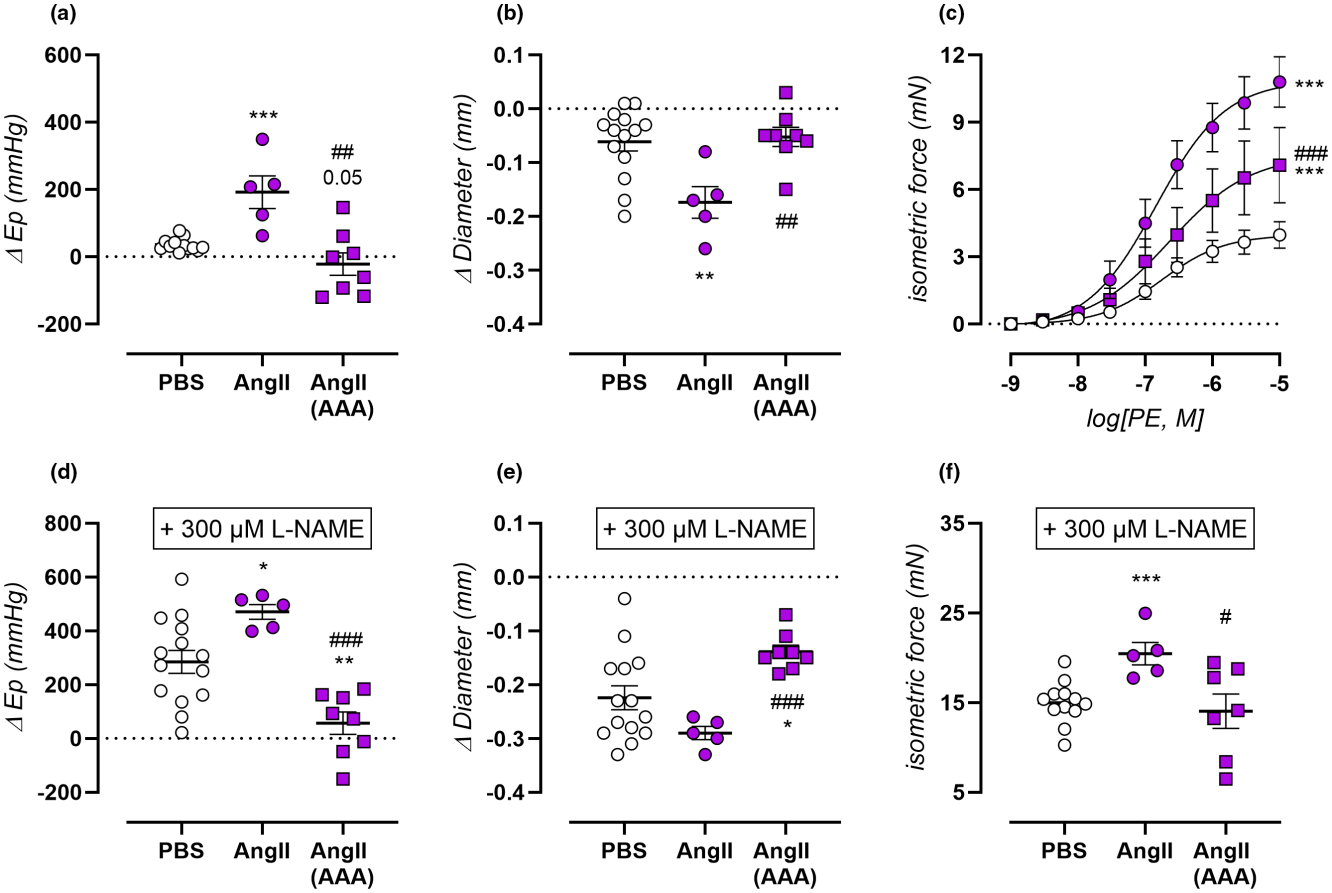
Effect of α_1_‐adrenergic contraction on aortic stiffness, diameter and isometric force in AngII‐treated mice at physiological pressures. Vasoactive responses of isolated aortic rings were measured for PBS‐treated control mice (*n* = 14, open circles), non‐aneurysmal 4‐week AngII‐treated mice (*n* = 5, filled circles), and aneurysmal 4‐week AngII‐treated mice (*n* = 8, filled squares). Aortic contraction was induced by 2 μM PE (a,b), concentration‐response PE (c) stimulation, or maximally contracted by 2 μM PE and 300 μM L‐NAME (d–f). Response of aortic contraction was measured on isobaric aortic stiffness at 80–120 mmHg (a,d), isobaric aortic diameter at 100 mmHg (b,e), and isometric force (c,f). Data are listed as mean ± SEM (c; *n* = 14, 5, 8) or each symbol represents a biological repeat (a,b,d–f). Statistical analysis using multiple *t*‐testing (a,b,d–f) or multiple two‐way ANOVA (c). Significance level compared to the PBS group is listed using *, and between AngII and AngII AAA groups using #. *T*‐test/overall two‐way ANOVA significance (in graph) are listed. */#*p* < 0.05, **/##*p* < 0.01, ***/###*p* < 0.001.

The effect of aortic contraction on isobaric aortic stiffness was assessed by addition of 2 μM PE to the organ bath. In non‐aneurysmal AngII‐treated mice, a heightened response was observed, both as E_p_ increase and diameter constriction (Figure 2a,b). In aneurysmal AngII‐treated mice however, no contraction‐dependent stiffening was observed (Figure 2a), although diameter recordings clearly show PE‐induced diameter constriction similar to control values (Figure 2b). This indicates that VSMC contraction was present but could not increase aortic stiffness beyond baseline values. Isometric contraction recordings during concentration‐response stimulation even revealed an elevated contractile response to PE in both aneurysmal and non‐aneurysmal mice (Figure 2c). In non‐aneurysmal mice, the maximal effect of PE‐induced contraction was the highest, reaching 10.1 ± 1.2 mN (*p* < 1.00E‐15) contraction with preserved sensitivity (EC_50_: −6.8 ± 0.1 log[M], *p* = 0.79). In aneurysmal AngII‐treated mice, contractions were slightly attenuated to 8.4 ± 1.6 mN although still significantly increased (*p* = 3.41E‐05) compared to those in PBS‐treated mice (4.4 ± 0.5 mN). A slight trend towards desensitization was also observed in aneurysmal AngII‐treated mice (EC_50_: −6.5 ± 0.2 log[M], *p* = 0.23).

In the presence of NOS blocker L‐NAME (300 μM) the contraction‐inhibitory effect of basal NO release was blocked, resulting in the induction of maximal PE‐induced contraction. In non‐aneurysmal AngII‐treated mice, this resulted in significantly increased aortic stiffening, with a trend towards increased diameter constriction (*p* = 0.11), and heightened isometric force development (Figure 2d–f). In aneurysmal AngII‐treated mice on the other hand, no aortic stiffening was observed due to maximal PE‐induced contraction, diameter constriction was present although significantly attenuated compared to PBS values, and maximal isometric force was unchanged (Figure 2d–f).

### 3.5 At calculated 120–160 mmHg pressure, contraction‐dependent stiffening is elevated in non‐aneurysmal and reversed in aneurysmal AngII‐treated mice

**FIGURE 3:**
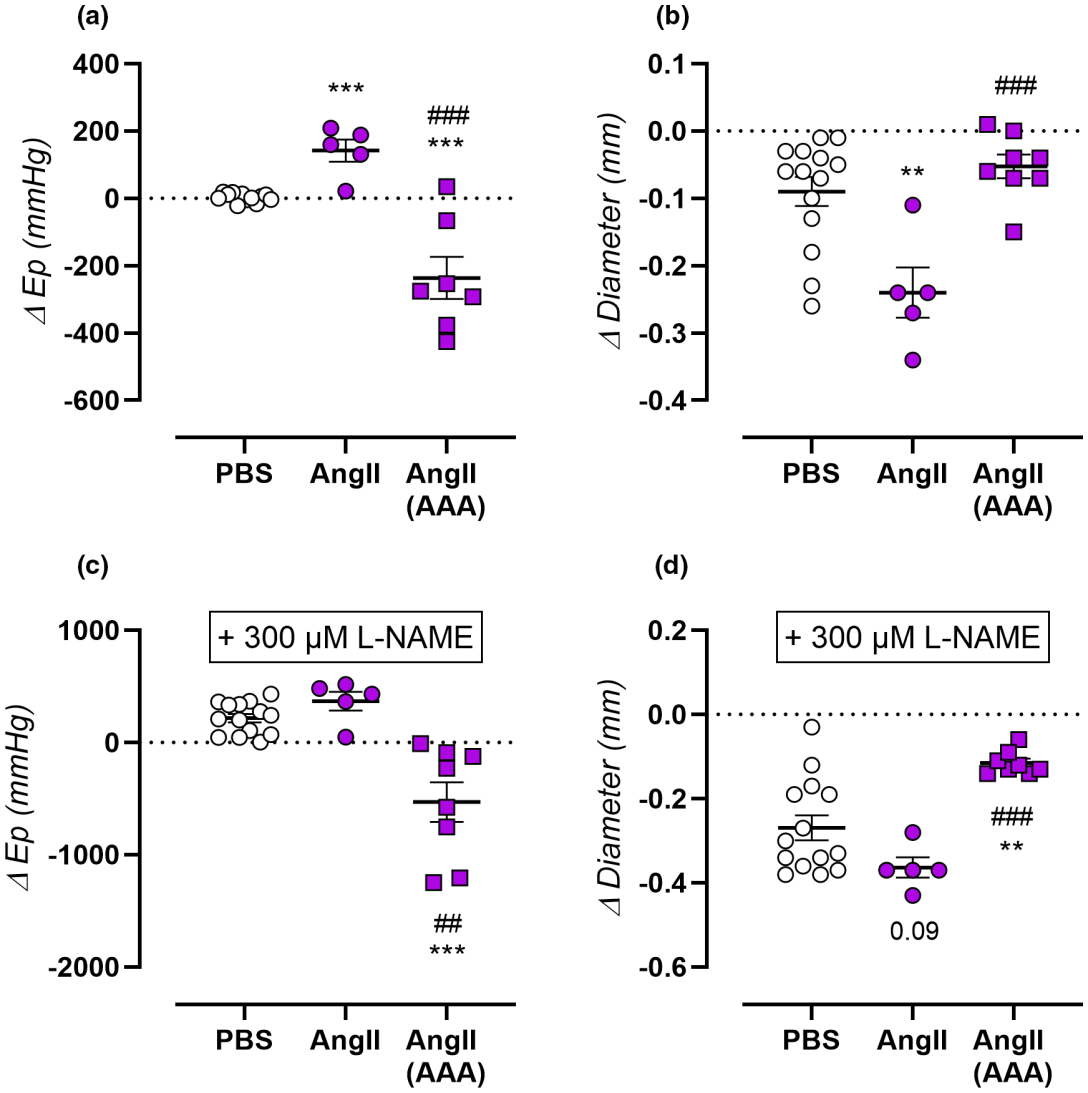
Effect of α_1_‐adrenergic contraction on aortic stiffness and diameter in AngII‐treated mice at high distending pressure. Vasoactive responses of isolated aortic rings were measured for PBS‐treated control mice (*n* = 14, open circles), non‐aneurysmal 4‐week AngII‐treated mice (*n* = 5, filled circles), and aneurysmal 4‐week AngII‐treated mice (*n* = 8, filled squares). Aortic contraction was induced by 2 μM PE in the absence (a,b) or presence (c,d) of 300 μM L‐NAME. Response of aortic contraction was measured on isobaric aortic stiffness at 120–160 mmHg (a,c) and isobaric aortic diameter at 160 mmHg (b,d). Each symbol represents a biological repeat. Statistical analysis using multiple *t*‐testing. Significance level compared to the PBS group is listed using *, and between AngII and AngII AAA groups using #. Significance is listed in graph. **/##*p* < 0.01, ***/###*p* < 0.001.

At high distending pressure, contraction‐dependent stiffening is inversed between aneurysmal and non‐aneurysmal AngII‐treated mice. Due to the pronounced AngII‐induced aortic stiffening at higher distending pressure, the effect of contraction on aortic stiffness and diameter was further assessed at calculated pressures of 120–160 mmHg. Interestingly, active aortic stiffening due to 2 μM PE stimulation was absent in PBS‐treated control mice. In non‐aneurysmal AngII‐treated mice, however, clear aortic stiffening was observed, whereas in aneurysmal AngII‐treated mice, pronounced destiffening was observed due to aortic contraction (Figure 3a). At a calculated pressure of 160 mmHg, the effect of AngII treatment on 2 μM PE‐induced aortic diameter constriction was similar to physiological 100 mmHg pressure, i.e., increased aortic constriction in non‐aneurysmal versus preserved aortic constriction in aneurysmal AngII‐treated mice (Figure 3b). In the absence of basal NO (after addition of 300 μM L‐NAME), PE‐induced aortic stiffening (diameter decrease) in non‐aneurysmal AngII‐treated mice and PE‐induced aortic destiffening (diameter decrease smaller than in PBS treated mice) in aneurysmal AngII‐treated mice were similar but had larger amplitudes (Figure 3c,d).

### 3.6 AngII‐treatment induces a pronounced desensitization of isometric relaxations to endogenously produced NO

2 μM PE‐precontracted aortic rings were exposed to concentration‐response stimulation with ACh to provoke endogenous endothelial NO production. Isometric relaxations revealed a desensitization in both AngII‐treated groups (Figure S5a), which was most pronounced in aneurysmal animals (IC_50_: AngII −7.18 ± 0.08 log(M), *p* = 0.05; AngII (AAA) −6.94 ± 0.11 log(M), *p* = 8.55E‐04) (Figure S5b). Maximal ACh‐induced relaxations were unchanged (Figure S5c). To assure that the above‐mentioned effects reflect alterations on the endothelial cell level, VSMC response to exogenous NO donor stimulation were tested by concentration‐response stimulation with DEANO. No significant changes were observed between groups (Figure S5d−f).

### 3.7 AngII‐treatment results in impaired basal NO production, leading to heightened VGCC contribution and basal VSMC contractile cytoplasmic calcium load

**FIGURE 4:**
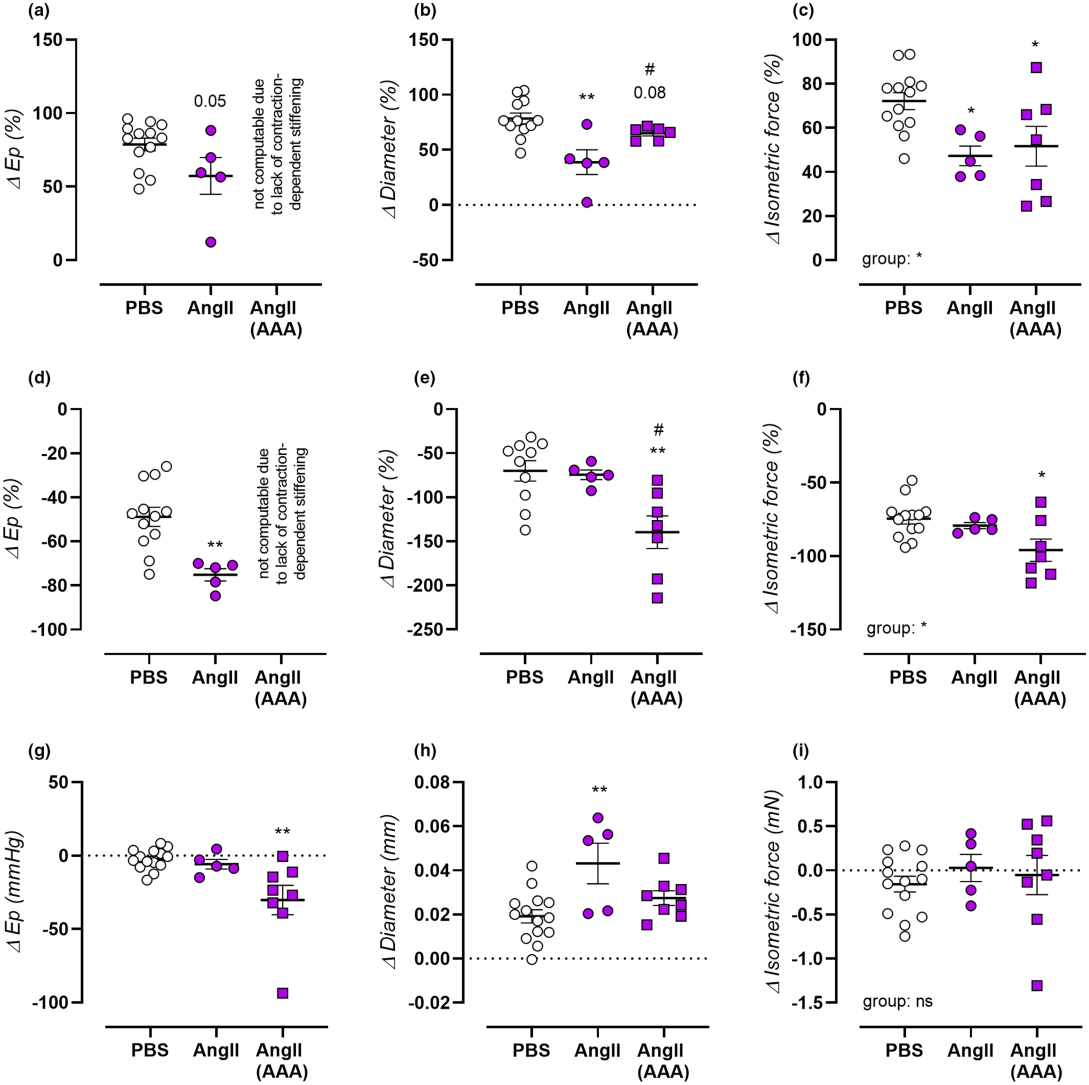
Assessment of basal NO levels, VGCC contribution, and basal VSMC tone in AngII‐treated mice. Vasoactive responses of isolated aortic rings were measured for PBS‐treated control mice (*n* = 14, open circles), non‐aneurysmal 4‐week AngII‐treated mice (*n* = 5, filled circles), and aneurysmal 4‐week AngII‐treated mice (*n* = 8, filled squares). Vasoreactivity was assessed as isobaric aortic stiffening at 80–120 mmHg (a,d,g), isobaric aortic diameter change at 100 mmHg (b,e,h), and isometric force development (c,f,i). Basal NO levels (a–c) were quantified as the difference in effect of 2 μM PE with without addition of 300 μM L‐NAME. The contribution of VGCC (d–f) was ascertained by addition of 35 μM diltiazem in 2 μM PE‐precontracted aortic rings. Basal VSMC tone (g–i) was assessed as the effect of replacement of normal Krebs ringer with 0Ca Krebs solution. Relative measurements of basal NO and VGCC contribution could not be computed as E_p_ change (a,d) in the AngII (AAA) group, since active contraction‐dependent aortic stiffening (denominator of fraction) in this group approximated 0. Each symbol represents a biological repeat. Statistical analysis using one‐way ANOVA (c,f,i) or (multiple) *t*‐testing (a,b,d,e,g,h). Significance level compared to the PBS group is listed using *, and between AngII and AngII AAA groups using #. Overall one‐way ANOVA significance (bottom) or post‐hoc/*t*‐test significance (in graph) are listed. Ns *p* > 0.05, */#*p* < 0.05, **p < 0.01.

Basal NO levels were assessed as the relative difference in effect of 2 μM PE in the presence and absence of NOS‐blocker L‐NAME. In non‐aneurysmal AngII‐treated mice, reduced basal NO levels were observed as reflected by E_p_, diameter, and isometric reactivity (Figure 4a−c). Basal NO levels were also reduced in aneurysmal AngII‐treated mice, although this effect was slightly attenuated compared to the non‐aneurysmal group. As a consequence, a heightened relative contribution of VGCC was also observed in both groups (Figure 4d−f). This was measured by addition of 35 μM diltiazem to 2 μM PE‐preconstricted aortic rings. The heightened effect of diltiazem was more pronounced in aneurysmal AngII‐treated mice, only reaching statistical significance in non‐aneurysmal AngII‐treated mice as an increased VGCC contribution to PE‐induced contraction‐dependent aortic stiffening (Figure 4d). By replacing normal Krebs‐Ringer environment with a 0Ca Krebs solution and thus avoiding basal calcium influx, a new equilibrium is formed for basal intracellular calcium and vessel tone. In PBS‐treated control mice, the effect of 0Ca Krebs replacement only differed from zero in the measurement of diameter (Figure 4h, possible time‐effect), but not for E_p_ or isometric force (Figure 4g,i), indicating low cytoplasmic free calcium levels in a healthy mouse aorta. In non‐aneurysmal AngII‐treated mice, a heightened response to 0Ca Krebs replacement was observed as increased diameter dilation only, indicating mild basal VSMC calcium loading. For aneurysmal AngII‐treated mice on the other hand, a heightened response was observed both as aortic destiffening and diameter dilation (*p* = 0.10), suggesting a more pronounced basal VSMC calcium load and vessel tone. No effect was observed in either AngII‐treated groups in the isometric force measurement (Figure 4i).

### 3.8 Sarcoplasmic reticulum‐mediated contraction alterations occur in AngII‐treated mice

**FIGURE 5:**
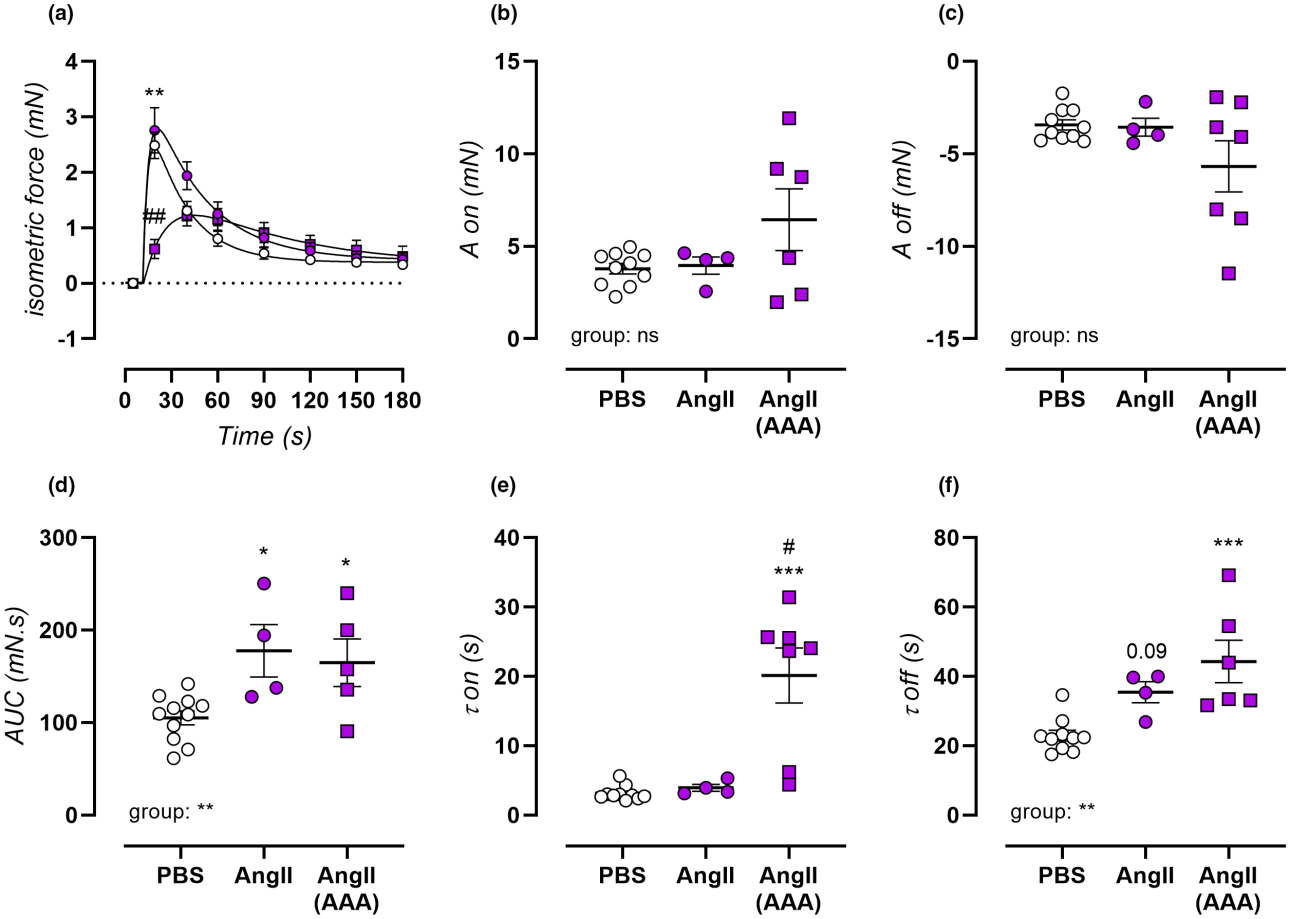
Transient SR‐mediated contraction alterations in AngII‐treated mice. Transient SR‐mediated contractions of isolated aortic rings were measured for PBS‐treated control mice (*n* = 14, open circles), non‐aneurysmal 4‐week AngII‐treated mice (*n* = 5, filled circles), and aneurysmal 4‐week AngII‐treated mice (*n* = 8, filled squares). A tracing of the transient SR‐mediated contraction was plotted (a), and the area curve was determined (d). Bi‐exponential fitting of the upward (on) and downward (off) phases of the contraction, amplitude (a) (b,c) and time constant (τ) (e,f) were calculated. Data are listed as mean ± SEM (a; *n* = 14, 5, 8) or each symbol represents a biological repeat (b–f). Statistical analysis using one‐way ANOVA (b–d,f) multiple *t*‐testing (e) or multiple two‐way ANOVA (a). Significance level compared to the PBS group is listed using *, and between AngII and AngII AAA groups using #. Overall one‐way ANOVA significance (bottom) or post‐hoc/*t*‐test/overall two‐way ANOVA significance (in graph) are listed. Ns *p* > 0.05, */#*p* < 0.05, **/##*p* < 0.01, ***p < 0.001.

In 0Ca Krebs solution, stimulation of aortic rings with 2 μM PE elicits a transient contraction resulting from IP_3_‐mediated calcium release from intracellular SR stores. A tracing of this contraction is plotted in Figure 5a, indicating larger SR‐mediated contractions in non‐aneurysmal, and smaller but broader SR‐mediated contractions in aneurysmal (non‐significant, *p* = 0.31) AngII‐treated mice. In both cases, this results in a significantly increased area under curve value (Figure 5d). Bi‐exponential fitting of the upward (contraction phase, on) and downward (relaxation phase, off) phases of this contraction was used to assess the amplitude (A) and time constant (τ) of each phase, revealing no significant changes in either amplitudes due to AngII‐treatment (Figure 5b,c). The time constant of phasic force increase (contraction phase), however, showed a marked increase in aneurysmal mice (Figure 5e). Moreover, the time constant of phasic force decrease (relaxation phase) displayed a non‐significant increase in non‐aneurysmal and extremely high value in aneurysmal AngII‐treated mice (Figure 5f). It can therefore be concluded that the large size of the SR‐mediated contraction in non‐aneurysmal AngII‐treated mice was mostly due to slow calcium removal, whereas the altered shape of SR‐mediated contraction of aneurysmal AngII‐mice resulted from reduced speed of both phases of the contraction.

### 3.9 AngII‐treatment induces VSMC phenotypic switch, migration and proliferation

**FIGURE 6:**
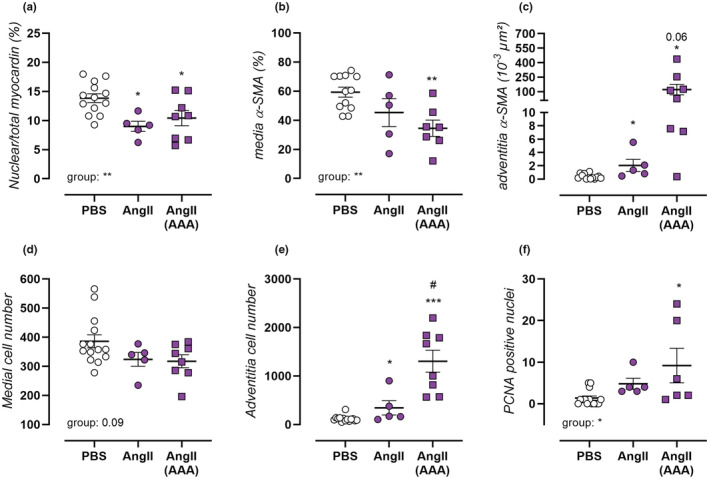
Angii‐treatment results in VSMC phenotypic switch, migration and proliferation. Histological analysis was performed on aortic tissue of PBS‐treated control mice (*n* = 14, open circles), non‐aneurysmal 4‐week AngII‐treated mice (*n* = 5, filled circles), and aneurysmal 4‐week AngII‐treated mice (*n* = 8, filled squares). IHC staining for myocardin was used to assess the nuclear/total myocardin fraction (a). IHC staining of VSMC‐marker, α‐smooth muscle Actin (α‐SMA) was used to assess media (b) and adventitia (c) area positivity. DAPI stained sections were used for counting media (d) and adventitia (e) cell number. And proliferating cell nuclear antigen (PCNA)‐positive cells were counted in the media (f). Each symbol represents a biological repeat. Statistical analysis using one‐way ANOVA (a,b,d,f) or multiple t‐testing (c,e). Significance level compared to the PBS group is listed using *, and between AngII and AngII AAA groups using #. Overall one‐way ANOVA significance (bottom) or post‐hoc/*t*‐test significance (in graph) are listed. */#*p* < 0.05, **p < 0.01, ***p < 0.001.

VSMC phenotype regulation was ascertained by histological measurement of myocardin localization, showing that AngII treatment resulted in a reduced nuclear myocardin fraction in the media, independent of AAA formation (Figure 6a). This indicates suppression of its transcriptional cofactor activity, and therefore loss of the regular VSMC contractile phenotype. Furthermore, a decreased percentage presence of medial VSMC was observed using staining of VSMC‐marker α‐SMA, which was non‐significantly reduced in non‐aneurysmal (*p* = 0.21) and strongly reduced in aneurysmal AngII‐treated mice (Figure 6b), and a non‐significant trend towards decreased medial cell count (Figure 6d). Interestingly, a concomitant increase in absolute α‐SMA positivity (Figure 6c) and cell count (Figure 6e) was noted in the adventitial layer. These effects were most pronounced in aneurysmal AngII‐treated mice, and indicate VSMC migration into the adventitia tissue layer. Finally, AngII‐treated mice also showed activation of cell proliferation, which only reached statistical significance in aneurysmal animals, measured as the number of PCNA positive cells in the media (Figure 6g). These data all confirm that AngII‐treatment induced a loss of the normal VSMC differentiation, followed by active VSMC proliferation and migration into the surrounding adventitia tissue layer. (Representative images, Figure S6).

## 4 DISCUSSION

A known effect of in vivo AngII‐treatment as an arterial aging model is the high likelihood of abdominal aorta aneurysm (AAA) formation, which involves progressive and irreversible focal dilation of the abdominal aorta that can lead to catastrophic rupture (Raffort et al., 2017). Incidence of AAA formation in AngII‐treated C57Bl6 mice can reach up to 75% (Trachet et al., 2015). Clinically, AAA formation develops silently and affects 1.2–3.3% of men over 60 years of age (Force USPST et al., 2019). Not only is aortic stiffness a known risk factor of AAA growth (Boczar et al., 2021), but adverse aortic connective tissue remodeling in response to AAA is a known contributor to aortic wall stiffening because of its association to inflammation (Wales et al., 2014), elastin degradation (Kavazos et al., 2015), and collagen restructuring (Jones et al., 2020). Furthermore, no pharmaceutical therapies have been shown to suspend or reverse AAA disease progression at this time (Force USPST et al., 2019). Hence, the spontaneous occurrence of AAA in AngII‐treated mice offers an interesting opportunity to investigate how AAA formation in AngII‐treated mice affects aortic function and biomechanics. In the present study, an exceptionally dissimilar aortic phenotype is reported in aneurysmal and non‐aneurysmal AngII‐treated mice. Cardiac hypertrophy and aortic stiffness are considerably more pronounced in aneurysmal AngII‐treated mice. Furthermore, ex vivo aortic reactivity studies showed that vasoactive agents diversely affect aortic biomechanical behavior in non‐aneurysmal and aneurysmal AngII‐treated mice. Since both aneurysmal and non‐aneurysmal AngII‐treated mice display a similar hypertensive phenotype, these differences are presumably blood pressure‐independent.

It is important to note, however, that the aortic rings for biomechanical testing were never obtained from aneurysm areas, since most aneurysms were located on the suprarenal abdominal aorta, and biomechanical testing was performed on proximal segments of the descending thoracic aorta. Thus, these differences reflect changes associated with the development of aneurysms at a different site on the aorta, rather than a direct comparison between aneurysmal and non‐aneurysmal aortic tissue.

### 4.1 Contraction‐dependent aortic stiffening at physiological and high distending pressure

The stiffness‐pressure relationship of the aorta is non‐linear (Leloup et al., 2019; Nichols & MacDonald, 2011), and ‐interestingly‐ remains remarkably conserved across all vertebrate and invertebrate species with a closed circulatory system (Shadwick, 1999), suggesting strong evolutionary pressure. Our research group (Leloup et al., 2019) previously demonstrated that maximal PE‐induced contraction increases aortic stiffness in the physiological pressure range, where elastin constitutes the predominant load‐bearing extracellular matrix (ECM) component. However, when mean aortic distending pressure increases above ~150 mmHg, wall stress is transferred to incompliant collagen fibers, and VSMC contraction was shown to attenuate aortic stiffness by disengaging collagen fiber alignment and shifting wall stress to VSMC and elastin fibers. This phenomenon is believed to constitute an important physiological regulatory system to maintain moderate aortic stiffness in periods of acute hypertension (e.g., exercise, stress), when catecholamine levels typically increase alongside blood pressure. A similar phenomenon was observed in the present study, where 2 μM PE induced‐aortic contraction in PBS‐treated control mice could increase aortic stiffness at physiological 80–120 mmHg pressures, but did not alter aortic stiffness at calculated 120–160 transmural pressures (around the turning point). In non‐aneurysmal AngII‐treated mice, however, significantly increased contraction‐dependent stiffening was observed in both conditions, suggesting that aortic wall restructuring has resulted in the inability of the aorta to alleviate its non‐linear elastic nature in this (broad) pressure range by modulation of VSMC contractile tone. In aneurysmal AngII‐treated mice on the other hand, the absence of contraction‐dependent aortic stiffness is already observed at physiological pressures, indicating that ECM stiffness has increased to such extent that it equals the structural cellular stiffness of VSMC in its contracted state. With increasing distending pressure, an extremely steep increase in aortic stiffness was observed in these mice, resulting in a significant destiffening at 120–160 mmHg distending pressure when VSMC contraction is initiated. These effects are thus related to the extreme passive aortic stiffness observed in aneurysmal AngII‐treated mice.

These results further underline the importance of investigating vasoactive arterial responses beyond classical isometric contraction studies, since heightened α_1_‐adrenergic contractions were measured in both non‐aneurysmal and aneurysmal AngII‐treated mice (in the absence of L‐NAME), but in both groups the heightened contractile response affects biomechanical behavior in a very dissimilar manner, and is therefore expected to diversely influence in vivo hemodynamics.

### 4.2 VSMC phenotypic switch, migration, and proliferation

In the present study, a decreased nuclear myocardin fraction was observed in medial VSMC of the aorta of both non‐aneurysmal and aneurysmal AngII‐treated mice. Myocardin is an essential cofactor of serum response factor (SRF), the transcription factor which initiates the transcription of many of the contractile VSMC phenotypic genes (e.g., Myh11, Acta2, Eln, Des, Vcl) (Miano, 2003). Myocardin is predominantly regulated by its subcellular location, as it undergoes cytoplasmic‐nuclear translocation upon activation (Li et al., 2003; Wang et al., 2003). As a result, functional differences in myocardin activity can be missed by classical reverse‐transcriptase PCR or Western blot testing since its regulation does not necessarily require transcription or translation. Therefore, nuclear/total myocardin levels were assessed in the current study by immunofluorescent staining of aortic sections, revealing nuclear export of myocardin in both AngII‐treated groups. In aneurysmal AngII‐treated mice, overall media myocardin level, α‐SMA level, and cell count were also decreased, which indicates substantial loss of VSMC in the cell media specifically in response to aneurysm formation. Since these changes were accompanied by a marked increase in adventitia α‐SMA level and cell count, it can be logically assumed that VSMC have migrated from the media to the adventitia, although loss of cell viability in the media was not investigated in the present study and thus cannot be excluded to be ‐ at least partially ‐ involved in the medial cell loss. AngII is a known stimulus for VSMC phenotypic switching (Huang et al., 2021), a process that is extensively studied in relation to cardiovascular disease, arterial stiffness and aging. This phenotypic switch is highly dependent on intracellular calcium handling (Matchkov et al., 2012), through the mechanism of excitation‐transcription coupling (Misarkova et al., 2016). In previous work of this research group, studying 1 week AngII‐treated mice, reduced basal NO availability led to increased basal contractile calcium level in the cytoplasm and an increased contribution of VGCC to adrenergic contractions (Leloup et al., 2018), similar to the findings in the current study after 4 week AngII treatment. These calcium signaling alterations might thus underly the aberrant VSMC phenotype induced by AngII. In the aneurysmal AngII‐treated mice of the present study, these changes resulted in a migratory VSMC phenotype, with loss of VSMC number in the media and pronounced adventitial remodeling.

Interestingly, the loss of contractile VSMC in the aortic media was accompanied with increased isometric α_1_‐adrenergic contractility. This might partially be explained by the increased presence of VSMC in the aortic adventitia, however, because of its different extracellular matrix and organization ‐ assuming that these VSMC are unlikely concentrically organized as in the medial layer ‐ it is unclear to which extent the adventitial VSMC could contribute to isometric force development or contraction‐dependent aortic stiffening. Alternatively, it might be argued that a loss in contractile VSMC phenotype does not necessarily lead to reduced contractility. Phenotypic switching has been described in a multitude of hypertensive animal models (Choi et al., 2019; Galmiche et al., 2013; Huang et al., 2021; Zhou, Lee, Stoll, Ma, Costa et al., 2017; Zhou, Lee, Stoll, Ma, Wiener, et al., 2017), which are usually also characterized by elevated contractile responses. Previous work by our research group also described that the loss of nuclear myocardin coincides with increased α_1_‐mediated contraction in spontaneously aging C57Bl/6 mice (De Moudt et al., 2022b). Therefore, this finding is not completely at odds with scientific consensus knowledge. A possible explanation for this phenomenon stems from the fact that a phenotypic switch is believed to result from excitation‐transcription coupling, a process in which calcium‐sensitive transcription factors alter gene expression in response to altered intracellular calcium levels (Misarkova et al., 2016). The wide array of calcium influx and efflux pathways in VSMC gives rise to numerous spatially and temporally regulated calcium signal variations, so that different calcium signaling patterns initiate distinct transcription events with diverse functional outcomes (Kudryavtseva et al., 2013). Since chronic exposure to high blood pressures has been shown to increase intracellular free calcium (Anwar et al., 2012), this could thus both induce a VSMC phenotype switch and increase contractility. This is consistent with the findings of the present study, to the extent that AngII‐treated mice displayed hypertension, VSMC phenotype alterations, and altered VSMC calcium handling (i.e., increased VGCC function, high VSMC cytoplasmic calcium load, altered intracellular contractile calcium store release from the SR, and slow contractile calcium export).

### 4.3 AngII‐treatment induced endothelial dysfunction

The present study describes pronounced endothelial dysfunction in AngII‐treated mice, including reduced sensitivity (IC_50_) to ACh‐induced NO production and decreased production of basal NO, without altered sensitivity of VSMC to exogenous NO donor DEANO. These data are in line with consensus scientific literature. Impaired endothelial relaxation is involved in blood pressure (Betrie et al., 2021; Panza et al., 1990; Perticone et al., 2001) and aortic stiffness (De Moudt et al., 2022a,c; Leloup et al., 2018) dysregulation, and is a known risk factor for cardiovascular disease (Rajendran et al., 2013). Mollnau et al (2002) showed that AngII infusion decreased NO production in the aorta of Wistar rats. Although the mechanism of AngII‐induced endothelial dysfunction remains uncertain, described mechanisms of action include direct binding and inhibition by the AT1 receptor to membrane‐localized eNOS (Marrero et al., 1999), eNOS uncoupling and supervenient superoxide production (Mollnau et al., 2002; Rajagopalan et al., 1996), and upregulation of arginase‐1 leading to reduced substrate availability (Shatanawi et al., 2011). Furthermore, high concentrations of AngII have been shown to reduce Akt phosphorylation through AT2 receptor signaling in cultured endothelial cells (Kou et al., 2007). Akt phosphorylation is known to induce eNOS (Ser^1177^) phosphorylation (Fulton, 2016), and was previously described by our research group as an important stimulus for maintaining basal NO production (Van Langen et al., 2012). The impaired sensitivity to ACh‐induced NO production was most pronounced in aneurysmal AngII‐treated mice, whereas the impaired basal NO production was slightly attenuated compared to non‐aneurysmal AngII‐treated mice, indicating that underlying mechanisms might be different in aneurysmal and non‐aneurysmal AngII‐treated mice.

### 4.4 1‐week versus 4‐week AngII‐treated mice

As stated, our research group previously published the biomechanical alterations in mice after 1‐week AngII infusion (Leloup et al., 2018). 1‐week AngII‐treated mice displayed no change in blood pressure and only a slight non‐significant increase in aPWV. Body weight was not altered and no aneurysm formation was noted. Contrarily, after 4‐week AngII treatment, the phenotype was much more pronounced, with significantly increased SBP, DBP, and PP, reduced body weight, and an approximately 60% aneurysm prevalence. Ex vivo aortic studies after 1‐week AngII‐treatment revealed no difference in diastolic diameter at calculated 80 mmHg pressure and a significant ~25% increase in E_p_ at calculated 80–120 mmHg pressure. In the present study, diastolic diameter (80 mmHg) was increased in aneurysmal AngII‐treated mice and unchanged in non‐aneurysmal AngII‐treated mice, and E_p_ (80–120 mmHg) was increased by ~6% and ~ 120% versus PBS‐treated control mice in non‐aneurysmal and aneurysmal AngII‐treated mice, respectively. Reactivity studies in 1‐week AngII treated mice revealed increased α_1_‐adrenoreceptor mediated aortic stiffening and isometric contractions, thereby resembling the effects in non‐aneurysmal 4‐week AngII‐treated mice. Impaired basal NO production and increased basal VSMC calcium load were observed in both studies. This confirms that many of the underlying aortic physiology changes described in this study were already present an earlier time point in the disease development, even though the pronounced systemic disease symptoms (i.e., weight loss, hypertension, elevated aPWV) had not yet developed. The present study additionally revealed distinct alterations in VGCC contribution, SR‐mediated transient contractions, ACh‐induced relaxations, pressure‐dependency in contraction‐mediated aortic (de)stiffening, and phenotypic, migratory, and proliferative state of medial VSMC, which were not previously investigated after 1‐week treatment. Therefore, it is impossible to deduce whether these changes occurred early or late in the disease development. Similarly, no significant aneurysm formation had occurred after 1‐week treatment, making it impossible to distinguish early‐onset disease characteristics between aneurysmal and non‐aneurysmal AngII‐treated mice.

Taken together, it can thus be concluded that, although aneurysmal and non‐aneurysmal 4‐week AngII‐treated mice showed similar underlying changes in aortic physiology (i.e., endothelial dysfunction, heightened α_1_‐adrenergic contractility, and altered VSMC calcium signaling), the biomechanics of the aorta were dissimilarly affected and in a markedly pressure‐dependent fashion, due to extreme passive aortic stiffening in aneurysmal AngII‐treated mice.

## AUTHOR CONTRIBUTIONS

SDM and PF were responsible for the conception and design of the work. Data was collected by SDM and JH. Data was analyzed and interpreted by SDM and PF. SDM drafted the article under the supervision of PF. Critical revision of the article was the responsibility of PF, WM, and GM. All authors approved the final version of the article.

## FUNDING INFORMATION

This work was supported by the University of Antwerp (GOA‐BOF, grant 33,931) and the Hercules Foundation (grant N° AUHA/13/03).

## CONFLICT OF INTEREST

None declared.

## Supporting information


Table S1
Figure S1
Figure S2
Figure S3
Figure S4
Figure S5
Figure S6
