# Supplementary material for: Disparate biomechanical properties of the aorta in non‐aneurysmal and aneurysmal mice treated with angiotensin II
Source: Physiol Rep. 2022 Sep 18;10(18):e15410. doi: 10.14814/phy2.15410 (PMC9483617; doi:10.14814/phy2.15410)
Supplement: Supplementary file 1 — Table S1 Figure S1 Figure S2 Figure S3 Figure S4 Figure S5 Figure S6 [file PHY2-10-e15410-s001.docx]

Disparate biomechanical properties of the aorta in non-aneurysmal and aneurysmal mice treated with angiotensin II

Sofie De Moudt, MSc;^1^* Jhana O. Hendrickx, MSc;^1^ Guido R.Y. De Meyer, PharmD, PhD;^1^ Wim Martinet, PhD;^1^ Paul Fransen, PhD^1^

^1^ Laboratory of Physiopharmacology, University of Antwerp, Belgium

***Corresponding author:**

Sofie De Moudt

[sofie.demoudt@uantwerpen.be](mailto:sofie.demoudt@uantwerpen.be)

Campus Drie Eiken T2.26, Universiteitsplein 1, 2620 Wilrijk

**Short title:** Aorta biomechanics of angiotensin II-treated mice

**Supplementary material**

## Supplemental Tables

**Table S1: AngII-treatment induces pronounced cardiac hypertrophy and fibrosis.**

|  | PBS (n=14) | AngII (n=5) | | AngII (AAA) (n=8) | | |
| --- | --- | --- | --- | --- | --- | --- |
| Heart weight/BW (%) | 0.53±0.01 | 0.62±0.01 | *** | 0.72±0.01 | *** | ## |
| IVS (mm) | 1.10±0.05 | 1.28±0.16 | ns | 1.53±0.15 | ** | ns |
| LVPW (mm) | 0.93±0.04 | 1.18±0.03 | ** | 1.19±0.13 | * | ns |
| LV mass/BW (%) | 0.39±0.02 | 0.64±0.05 | *** | 0.73±0.07 | *** | ns |
| SV (µL) | 37.3±2.4 | 40.0±1.7 | ns | 43.0±2.7 | ns | ns |
| EF (%) | 72.9±2.5 | 63.3±6.2 | ns | 71.4±3.7 | ns | ns |
| LVID (mm) | 3.51±0.11 | 3.87±0.12 | ns | 3.78±0.16 | ns | ns |
| E/A ratio | 1.6±0.2 | 1.4±0.3 | ns | 1.8±0.1 | ns | ns |
| IVRT (ms) | 20.9±1.9 | 20.5±2.4 | ns | 22.5±3.5 | ns | ns |
| Deceleration (ms) | 17.7±2.3 | 17.9±2.9 | ns | 18.4±3.0 | ns | ns |
| Cardiac fibrosis area (%) | 0.89±0.17 | 2.91±0.44 | *** | 2.39±0.54 | ** | ns |
| Cardiomyocyte area (µm²) | 318±8 | 421±18 | *** | 439±14 | *** | ns |

Heart and body weight were recorded at time of sacrifice. Cardiac fibrosis area and cardiomyocyte cross-sectional area were ascertained histologically by use of Trichrome Masson and laminin staining respectively. Other parameters were measured by echocardiography. Abbreviations: A, atrial contraction wave; BW, body weight; E, early wave; EF, ejection fraction; IVRT, isovolumic relaxation time; IVS, interventricular septum; LV, left ventricle; LVID, LV internal diameter; LVPW, LV posterior wall; SV, stroke volume. Data are listed as mean±SEM. Statistical analysis using multiple t-testing between PBS versus AngII and PBS versus AngII (AAA) groups. T-test significance is listed in the table with significance level compared to the PBS group listed using *, and between AngII and AngII AAA groups using #. ns p>0.05, * p<0.05, **/## p<0.01, *** p<0.001.

## Supplemental Figures and Figure Legends

**
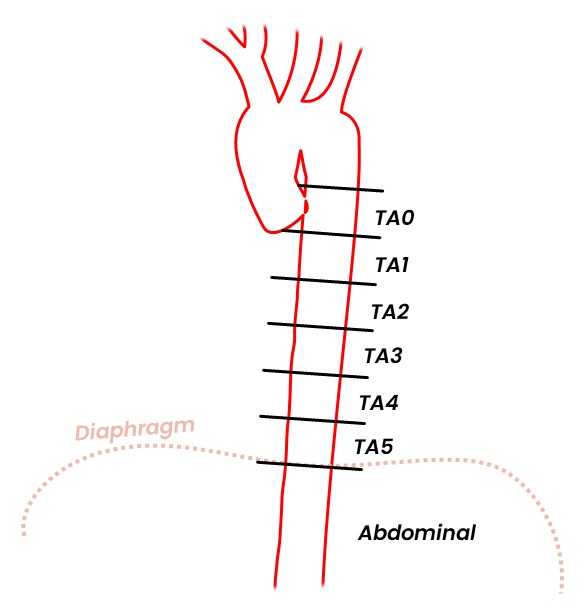
**

**Figure S1: Methodological diagram of aortic sectioning.** Using the diaphragm as an anatomical reference point, 2-mm aortic rings were cut from the descending thoracic aorta starting at the diaphragm in the distal to proximal direction. Aortic rings were numbered TA0 to TA5.

**
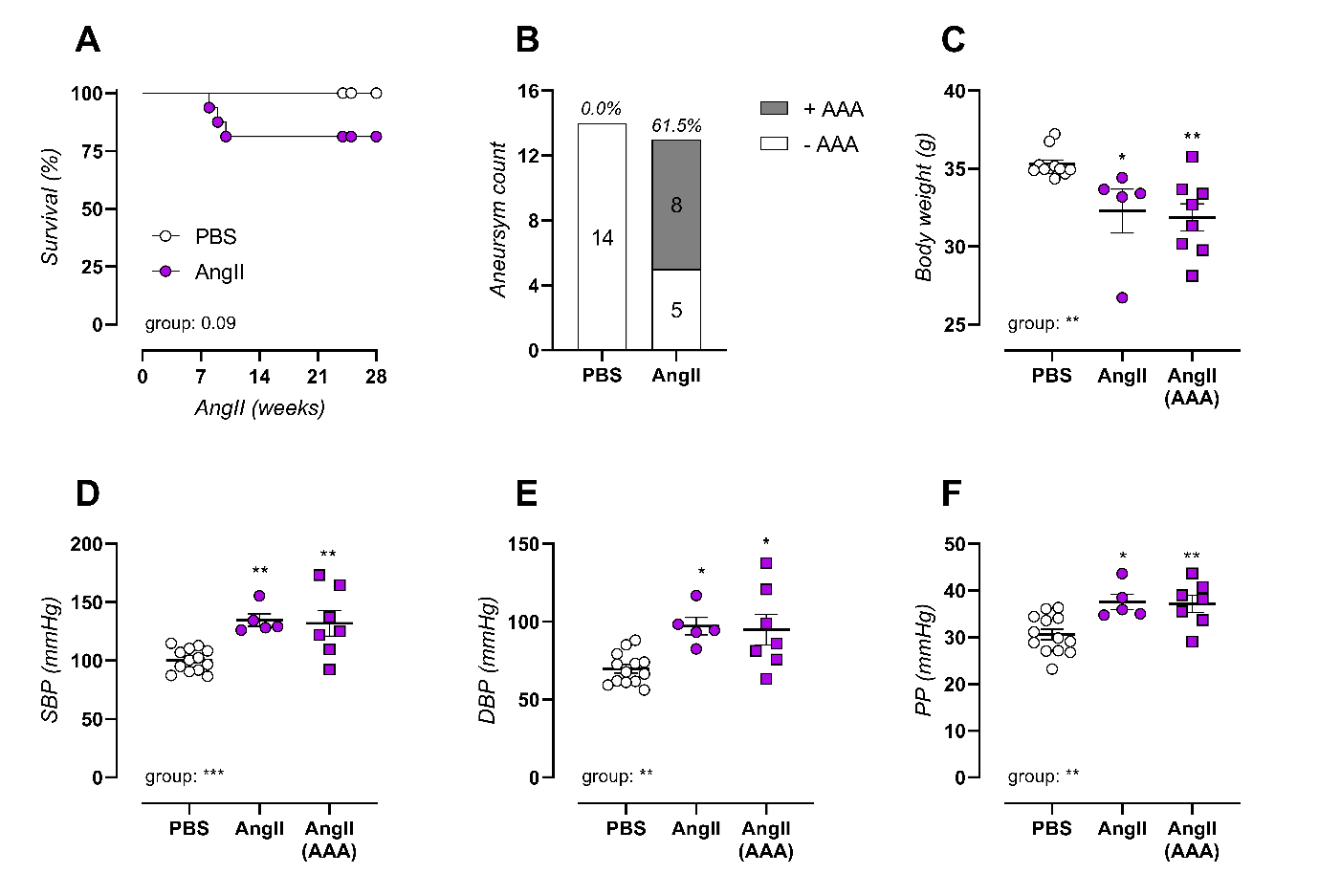
**

**Figure S2: Survival, aneurysm formation, body weight and peripheral blood pressure of AngII-treated mice.** Measurements were performed in PBS-treated control mice (n=14, open circles), non-aneurysmal 4-week AngII-treated mice (n=5, filled circles), and aneurysmal 4-week AngII-treated mice (n=8, filled squares). Survival analysis (A) and occurrence of abdominal aorta aneurysm (AAA, B) were plotted. Measurement of body weight (C) and peripheral systolic blood pressure (D), diastolic blood pressure (E), and pulse pressure (F) were stratified depending on the presence of an AAA due to AngII treatment. Each data point represents an individual biological repeat (n>5). Statistical analysis was performed using Mantel-Cox test (A) or one-way ANOVA testing (C-F). Overall (bottom) and post-hoc (in graph) significance are listed for testing versus PBS group. * p<0.05, ** p<0.01, *** p<0.001. No significant changes in post-hoc testing between AngII and AngII AAA groups were observed.
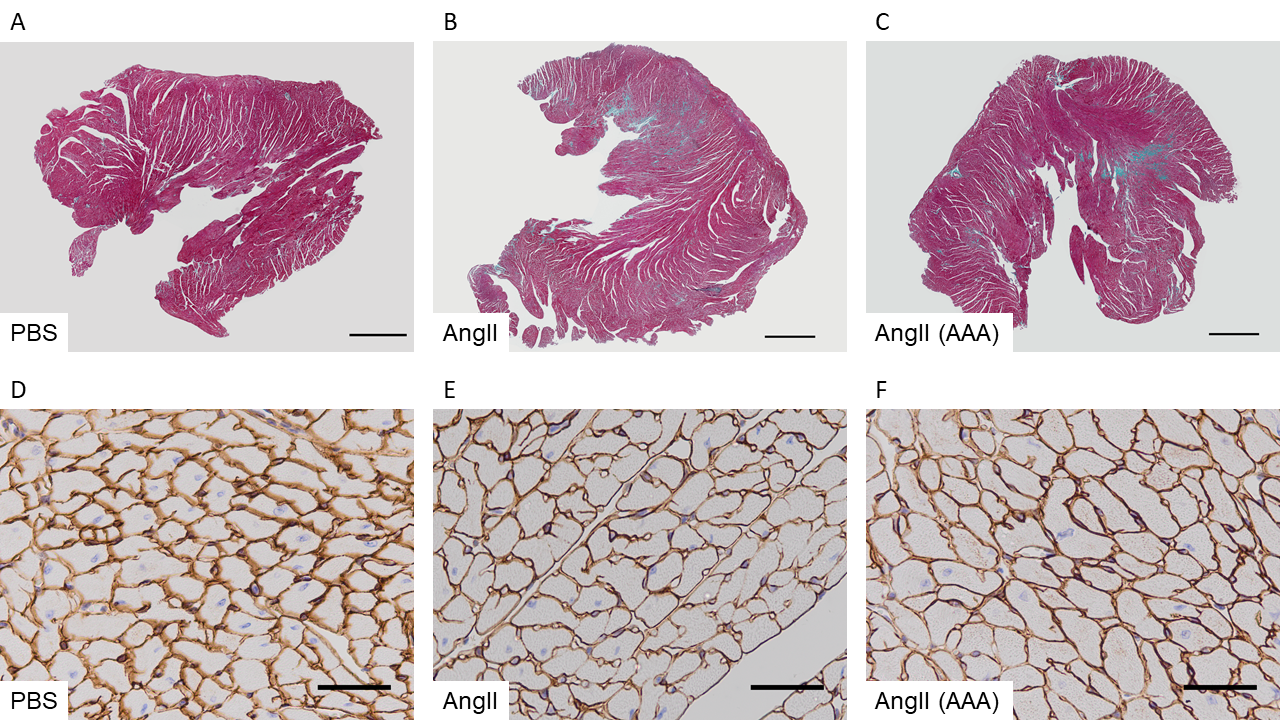


**Figure S3: Histological assessment of interstitial cardiac fibrosis and cardiomyocyte hypertrophy.** Representative image of trichrome Masson stained cardiac sections for assessment of fibrosis in PBS-treated mice (A), non-aneurysmal AngII-treated mice (B) and aneurysmal AngII-treated mice (C). Representative image of laminin stained cardiac sections for assessment of cardiomyocyte cross sectional area in PBS-treated mice (D), non-aneurysmal AngII-treated mice (E), and aneurysmal AngII-treated mice (F). Scale bars represent a distance of 1 mm (A-C) or 50 µm (D-F).


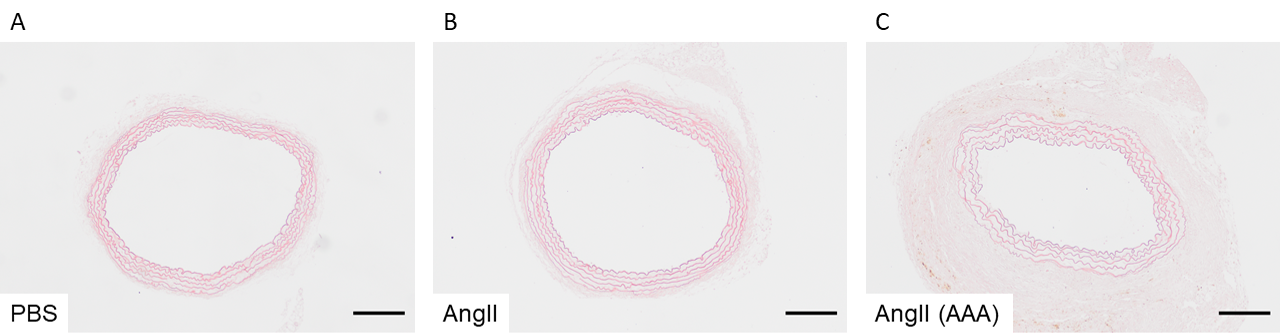


**Figure S4: Histological assessment of thoracic aorta elastin content.** Representative image of orcein stained thoracic aortic sections for assessment of wall thickness, elastin positive area, elastin break count, and elastin laminae count in PBS-treated mice (A), non-aneurysmal AngII-treated mice (B), and aneurysmal AngII-treated mice (C). Scale bars represent a distance of 200 µm.


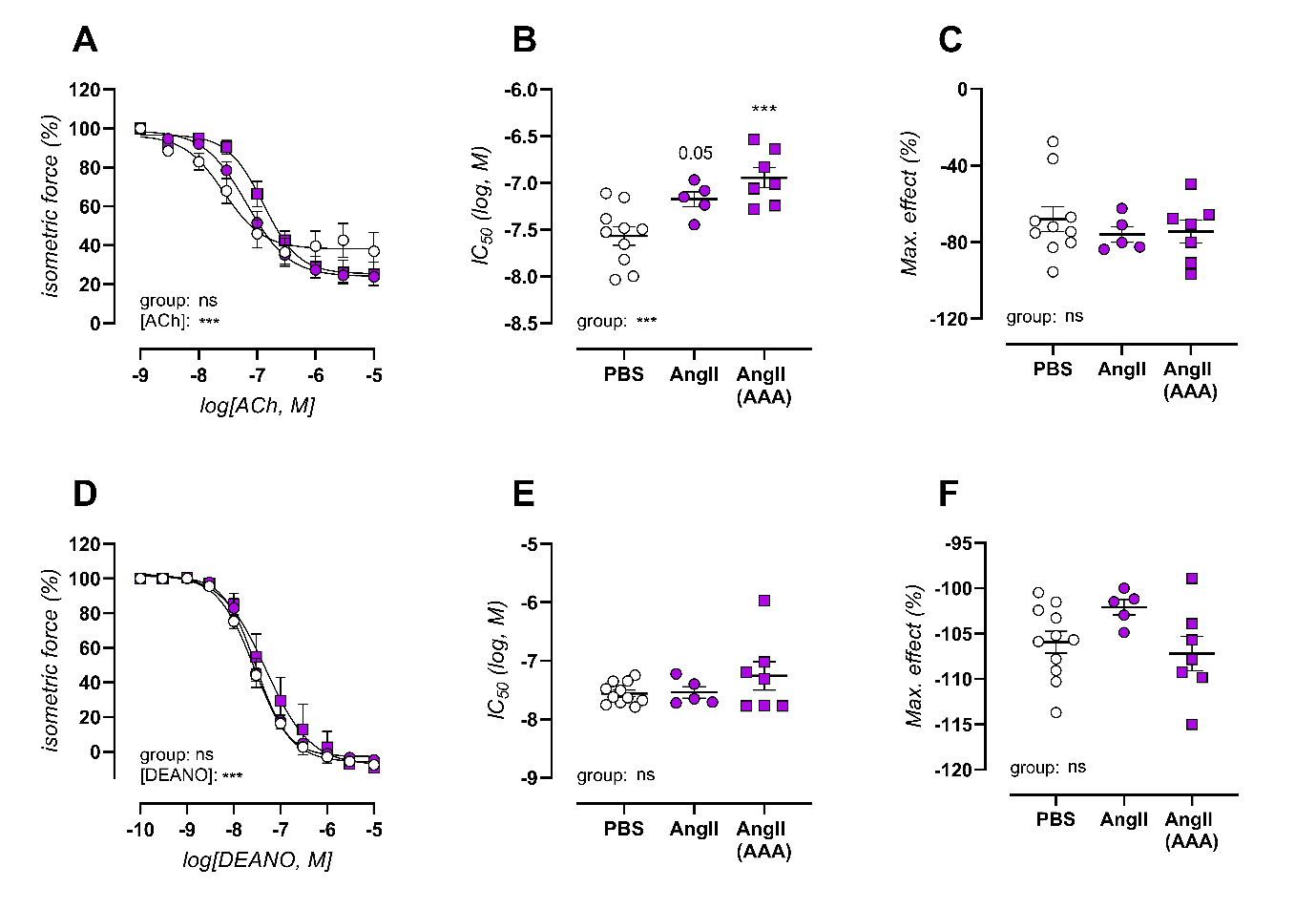


**Figure S5: AngII-treatment results in a desensitization of ACh-induced aortic relaxations.** Vasoactive responses of isolated aortic rings were measured for PBS-treated control mice (n=14, open circles), non-aneurysmal 4-week AngII-treated mice (n=5, filled circles), and aneurysmal 4-week AngII-treated mice (n=8, filled squares). Isometric relaxations were studied by concentration-response stimulation with ACh in 2 µM PE-precontracted aortic rings (A-C), and concentration-response stimulation with DEANO in 2µM PE + 300 µM L-NAME-precontracted aortic rings (D-F). Of each assay, relative concentration-response curves are shown (A,D), which were fitted with a non-linear 4‑parameter equation to calculate IC_50_ (B,E) and maximal effect (C,F). Data are listed as mean±SEM (A,D; n=14, 5, 8) or each symbol represents a biological repeat (B-C,E-F). Statistical analysis using two-way (A,D) or one-way (B-D,E-F) ANOVA. Overall ANOVA significance (bottom) and post-hoc significance (in graph) are listed. ns p>0.05, *** p<0.001.


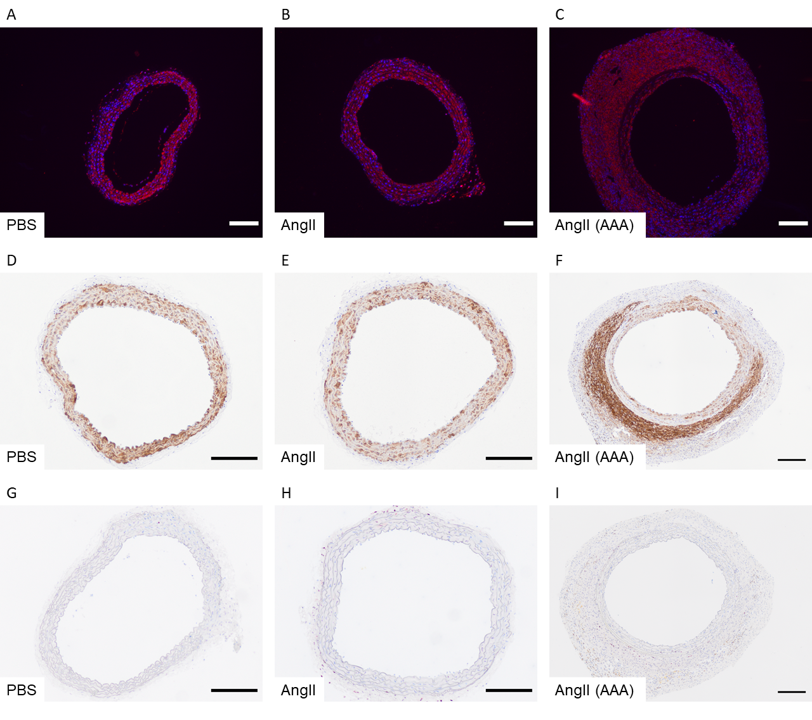


**Figure S6: Histological assessment of VSMC phenotypic switch, migration, and proliferation.** Representative image of myocardin (red) and DAPI (nuclear staining, blue) stained thoracic aortic sections for assessment of nuclear fraction myocardin and total media myocardin positive area in PBS-treated mice (A), non-aneurysmal AngII-treated mice (B), and aneurysmal AngII-treated mice (C). Representative image of α-smooth muscle actin (α -SMA, VSMC-marker) stained thoracic aortic sections for assessment of media and adventitia area positivity in PBS-treated mice (D), non-aneurysmal AngII-treated mice (E), and aneurysmal AngII-treated mice (F). Representative image of proliferation cell nuclear antigen (PCNA) stained thoracic aortic sections for assessment of proliferating cell count in the aortic media in PBS-treated mice (G), non-aneurysmal AngII-treated mice (H), and aneurysmal AngII-treated mice (I). Scale bars represent a distance of 200 µm.
